# Dataset showing the impact of the protonation states on molecular dynamics of HIV protease

**DOI:** 10.1016/j.dib.2016.07.040

**Published:** 2016-07-25

**Authors:** Rosemberg O. Soares, Pedro H.M. Torres, Manuela L. da Silva, Pedro G. Pascutti

**Affiliations:** a Instituto de Biofísica Carlos Chagas Filho (IBCCF), Universidade Federal do Rio de Janeiro (UFRJ), Rio de Janeiro, Brazil; b Diretoria de Metrologia Aplicada às Ciências da Vida (DIMAV), Instituto Nacional de Metrologia Qualidade e Tecnologia (INMETRO), Xerém, Brazil

## Abstract

The data described here supports the research article “Unraveling HIV Protease Flaps Dynamics by Constant pH Molecular Dynamics Simulations” (Soares et al., 2016) [1]. The data involves both standard Molecular Dynamics (MD) and Constant pH Molecular Dynamics (CpHMD) to elucidate the effect of protonation states of catalytic dyad on the HIV-PR conformation. The data obtained from MD simulation demonstrate that the protonation state of the two aspartic acids (Asp25/Asp25′) has a strong influence on the dynamics of the HIV-PR. Regarding the CpHMD simulation, we performed *pk_a_* calculations for HIV-PR and the data indicate that only one catalytic aspartate should be protonated.

**Specifications Table**

**Table:** 

Subject area	Biophysics, Biochemistry
More specific subject area	Molecular Dynamics Simulations
Type of data	Graph, figure and table
How data was acquired	Standard and constant pH molecular dynamics simulation of HIV-PR was performed using GROMACS 4.4 [2], GROMOS96 force field [3], Amber12 [4] and AMBERff99SB force field [5].
Data format	Analyzed
Experimental factors	The systems were minimization until reaching a gradient of 2.39 kcal mol^−1^ Ǻ^−1^ then equilibrated for 4 ns.
Experimental features	NPT ensemble at 300 K
Data source location	Institute of Biophysics Carlos Chagas Filho (IBCCF), Federal University of Rio de Janeiro (UFRJ), Rio de Janeiro, Brazil
Data accessibility	Data is within this article

**Value of the data**

• The present data demonstrate the importance of the protonation states in molecular simulations and provide new insights regarding HIV-PR flap conformation.
• These data findings might be useful to the selection of the protonation state in HIV-PR.
• The results highlight the importance of the constant pH molecular dynamics CpHMD technique.
• The CpHMD method used in this work provides new insights into the pH dependent activity of the HIV-PR.

## 1 Data

**Fig. 1:**
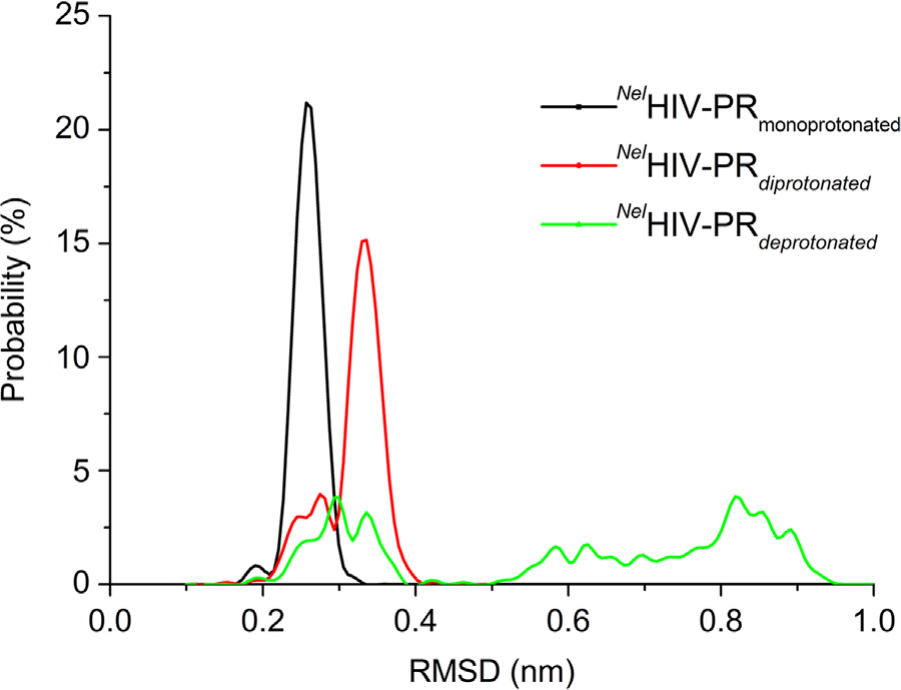
Comparison between the distribution of RMSD distance of all the pair of conformations of the trajectory of the deprotonated (green line), monoprotonated (black line) and diprotonated (red line) HIV-PR.

**Fig. 2:**
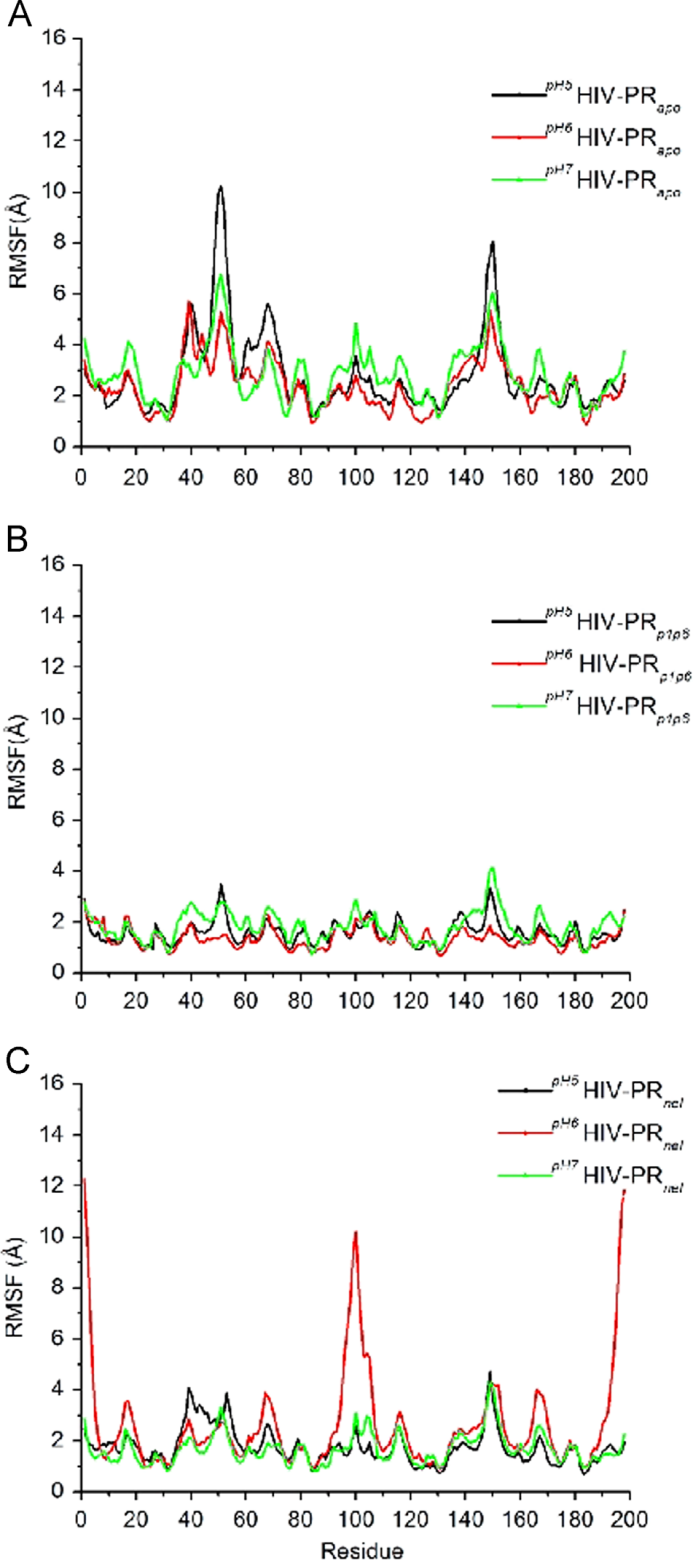
(A) HIV-PR protease backbone fluctuations at pH 5 (black line), 6 (red line) and 7 (green line). (A) Apo HIV-PR. (B) p1p6-bound HIV-PR. (C) nelfinavir-bound HIV. The chain A residues are numbered from 1-99 and 1’−99’ from chain B.

**Fig. 3:**
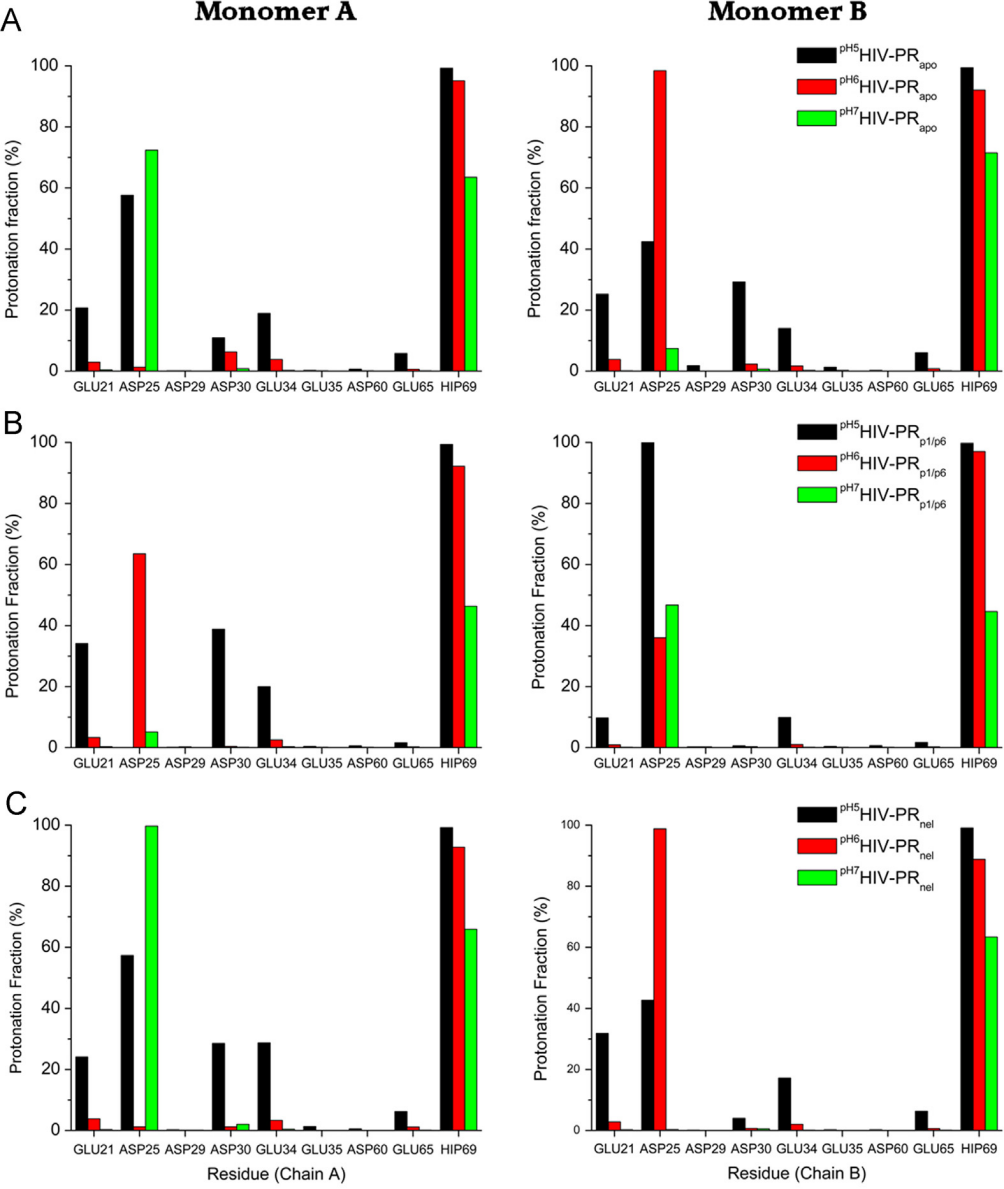
The protonation fraction of all Glu, Asp and His from apo (panel A), p1p6-bound (panel B) and nelfinavir-bound HIV-PR (panel C) at pH values of 5 (black bars), 6 (red bars) and 7 (green bars). The charts on the left represent the A chain, whilst the ones on the right represent the B chain.

**Fig. 4:**
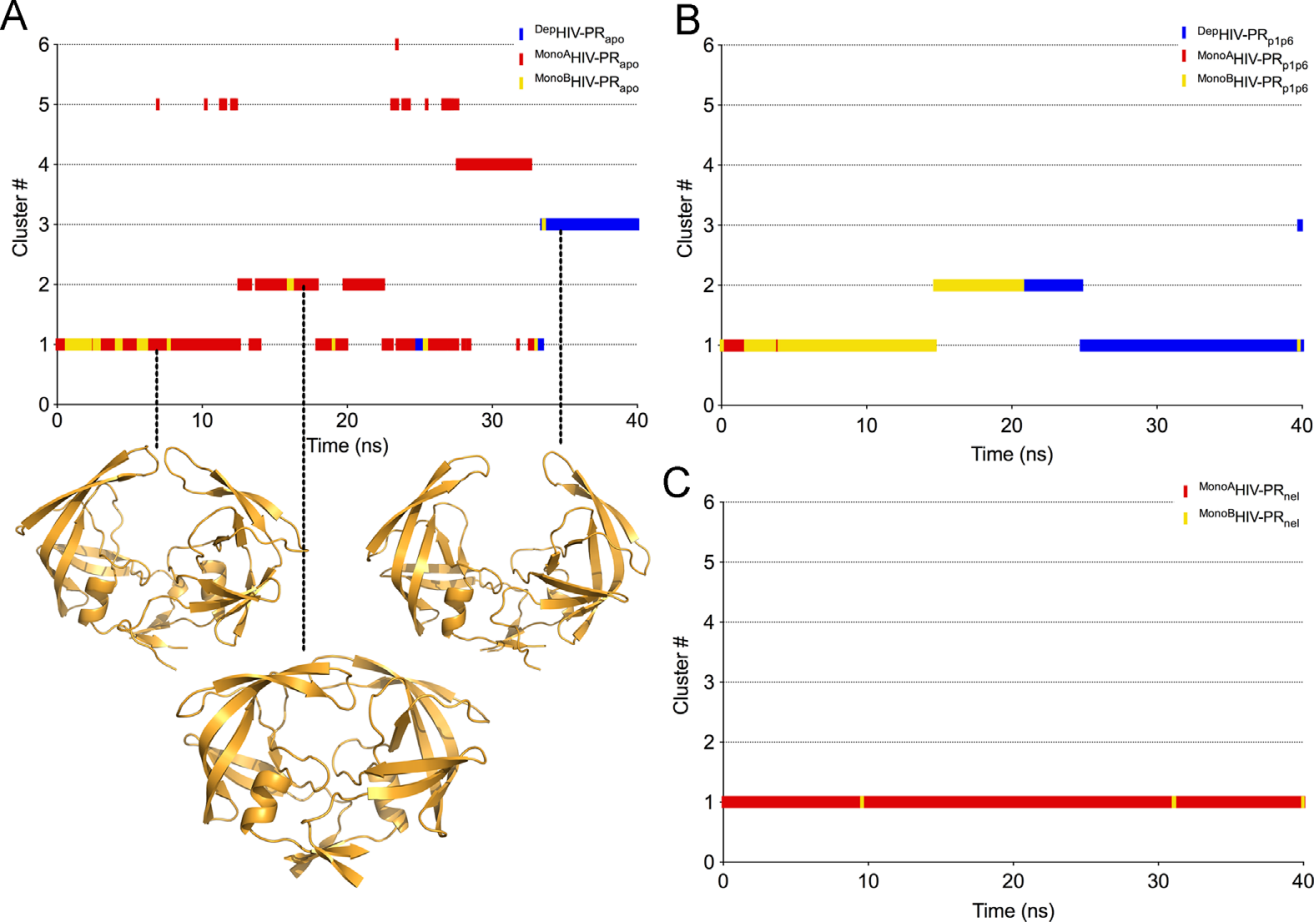
Correlation between the conformational and protonation states for apo (panel A), p1p6-bound (panel B) and nelfinavir-bound HIV-PR (panel C) at pH 7. The *y* axis represents the RMSD cluster number on the respective time frame (x axis). The blue, red and yellow bars represent deprotonated, protonation of Asp25 and protonation of Asp25׳, respectively. The Dashed lines point to the centroids of the three most prevalent clusters found throughout the simulation time of the apo system (clusters 1, 2 and 3). The clusters centroids of the remaining systems ("p1p6" and "nel") were not represented.

The data presented here comprise results obtained from MD and CpHMD to investigate the functional role of protonation states on HIV-PR. The analyses that were carried out employed mainly three systems: free HIV-PR, HIV-PR complexed with a natural substrate (p1p6 peptide) and HIV-PR complexed with an inhibitor compound (Nelfinavir). We have measured the root mean square deviation profile in different protonation states using standard MD simulation (Fig. 1), root mean square fluctuation in different pH values using CpHMD (Fig. 2, Table 1), protonation ratio of all ionizable residues (Fig. 3) and conformational cluster analysis (Fig. 4). Table 2 elucidates an unusually low predicted *pK_a_* for Glu35, through a hydrogen bond prevalence analysis.

## 2 Experimental design, materials and methods

### 2.1 Systems Setup

The atomic coordinate entries available in the PDB under accession codes 1OHR [6] and 1KJF [7] were used as initial condition of the NFV and p1/p6 bound-HIV-PR, respectively. The crystal structure 2HB4 was used as apo form of HIV-PR [8]. Before starting the simulation on the 1KJF structure, we performed the mutation N25D [1].

### 2.2 MD simulations

The nelfinavir-bound HIV-PR was simulated under three different protonation states in the catalytic residues: deprotonated (both residues are not protonated), monoprotonated (only the Asp25’ is protonated) and deprotonated (both residues are protonated). We performed MD simulations with the program package GROMACS v. 4.4, using the GROMOS96 (43A1) force field and the SPC water model [9]. The solvation procedure was performed with a layer of at least 15 Å around the complex (approximately 11,350 water molecules). Due to their net positive charge, an appropriate number of chloride counter-ions were added to neutralize the system. The topology for nelfinavir was taken from a previous reference [10]. For Coulomb interactions, the reaction field correction term [11] was employed, with Cutoff 1.4 Å and a dielectric constant set to 54 [12]. All systems were run in periodic boundary conditions and the NPT ensemble. The temperature was maintained at 300 K and pressure at 1 atm using the Berendsen weak coupling approach [13]. LINCS and SETTLE were applied to constrain solute and solvent bonds respectively. Each initial set up was optimized using three different steps. First, an energy minimization using the steepest-descent algorithm was made restraining the protein and ligand heavy atoms to their original positions with a harmonic potential of 10 kcal mol^−1^ Ǻ^−2^. Then, another minimization using steepest-descent with no restraints was performed. Finally, an energy minimization procedure with all restraints already removed was conducted, using the conjugate gradient method until reaching a gradient of 2.39 kcal mol^−1^ Ǻ^−1^. Following the minimization, two stages of equilibration were performed: a 500 ps of MD with protein non-hydrogen atoms positions restrained by harmonic potential of 2 kcal mol^−1^ Ǻ^−2^, in a NVT ensemble and other of 2 ns MD simulation with no position restraints, in a NPT ensemble. Finally, 60 ns of MD were conducted for further analysis.

### 2.3 CpHMD simulations

The HIV-PR was simulated in its apo form, and bound to p1p6 and nelfinavir at pH 5.0 pH 6.0 and pH 7.0 for 40 ns and 80 ns. The CpHMD simulations were performed with the program package Amber12 and the AMBER ff99SB force field [5]. The parameters for nelfinavir were obtained using the antechamber module and the GAFF force field [14]. All systems were run using implicit solvation model (igb=2) [15], [16], [17] under NVT ensemble. *Before the simulation only the Asp25’ was protonated.* For non-bonded interactions was employed a cutoff 30 Å. The system temperature was maintained at 300 K using the Berendsen thermostat [18]. All bond lengths involving hydrogen atoms were constrained using the SHAKE algorithm [19]. The same processes described in the *previous section were also employed in this step, all Glu, Asp and His residues were allowed to change protonation states (18 residues). Protonation state change attempts were made every 10 fs.*

## Figures and Tables

**Table 1:** Calculated *pK*_*a*_ values for residues from apo, p1p6 and bound-nelfinavir HIV-PR at pH 7 over 40 ns and 80 ns.

**Residue**	**Codon**	**HIV-PR**_***apo***_				**HIV-PR**_***p1/p6***_				**HIV-PR**_***Nel***_			
		**40 ns**		**80 ns**		**40 ns**		**80 ns**		**40 ns**		**80 ns**	
		**Chain A**	**Chain B**	**Chain A**	**Chain B**	**Chain A**	**Chain B**	**Chain A**	**Chain B**	**Chain A**	**Chain B**	**Chain A**	**Chain B**
**GLU**	**21/21′**	4.4	4.0	4.4	4.1	4.5	3.9	4.7	3.7	4.3	4.2	4.5	4.3
**ASP**	**25/25′**	7.2	5.7	6.6	5.4	5.7	6.9	5.4	6.5	9.3	4.3	9.6	4.0
**ASP**	**29/29′**	2.8	2.8	2.8	2.7	2.7	2.5	2.5	2.9	3.6	2.7	3.3	2.6
**ASP**	**30/30′**	4.7	4.6	4.7	4.5	4.1	3.3	3.8	3.8	5.1	4.5	4.9	4.3
**GLU**	**34/34′**	4.2	4.1	4.2	4.0	4.5	3.7	4.4	3.4	4.4	3.8	4.5	3.8
**GLU**	**35/35′**	2.8	2.3	2.8	3.0	3.4	2.5	3.2	2.4	3.2	2.8	3.0	2.7
**ASP**	**60/60′**	2.3	1.6	2.3	1.8	1.5	2.8	2.9	2.8	0.9	2.0	2.0	1.7
**GLU**	**65/65′**	3.8	3.8	3.7	3.8	3.2	2.9	3.2	3.0	3.6	3.8	3.6	3.7
**HIS**	**69/69′**	7.0	7.2	7.1	7.2	6.9	6.9	7.0	6.9	7.1	7.0	7.1	7.1

- (Dash) denotes that the *pk*_*a*_ value was not possible to be calculated, because there was no enough change in the protonation of residue.

**Table 2:** Prevalence (%) of hydrogen bond interactions between Glu 35 and Arg 57 during the simulation.

**System**	**Residues (atoms)**			**pH5**		**pH6**		**pH7**	
				**Chain A**	**Chain B**	**Chain A**	**Chain B**	**Chain A**	**Chain B**
**HIV-PR***_**free**_*	E35(OE1/OE2)	–	R57(NH1/NH2)	78	79	60	83	74	89
**HIV-PR***_**p1p6**_*	E35(OE1/OE2)	–	R57(NH1/NH2)	88	90	75	66	68	75
**HIV-PR***_**nel**_*	E35(OE1/OE2)	–	R57(NH1/NH2)	72	90	90	79	78	42

## References

[1] Soares R.O., Torres P.H.M., da Silva M.L., Pascutti P.G. (2016). Unraveling HIV protease flaps dynamics by constant pH molecular dynamics simulations. J. Biol. Struct..

[2] Hess B., Kutzner C., Van Der Spoel D., Lindahl E. (2008). GROMACS 4: algorithms for highly efficient, load-balanced, and scalable molecular simulation. J. Chem. Theory Comput..

[3] Van Gunsterem W.F., Billeter S.R., Eising A.A., Hünenberger P.H., Krüger P., Mark A.E., Scott A.E.X., Tironi I.G. (1996). Biomolecular Simulation: The GROMOS96 manual and User Guide.

[4] Case D.A., Darden T.A., Cheatham T.E., Simmerling C.L., Wang J., Duke R.E., Luo R., Walker R.C., Zhang W., Merz K.M., Roberts B., Hayik S., Roitberg A., Seabra G., Swails J., Götz A.W., Kolossváry I., Kolossváry K.F., Paesani F., Vanicek J., Wolf R.M., Liu J., Wu X., Brozell S.R., Steinbrecher, Gohlke T., Cai H., Ye Q., Wang X., Hsieh J., Cui M.-J., Roe G., Mathews D.R., Seetin D.H., Salomon-Ferrer M.G., Sagui R., Babin C., Luchko V., Gusarov T., Kovalenko S., Kollman, P.A A. (2012). AMBER 12.

[5] Hornak V., Abel R., Okur A., Strockbine B., Roitberg A., Simmerling C. (2006). Comparison of multiple amber force fields and development of improved protein backbone parameters. Proteins: Struct. Funct., Bioinforma..

[6] Kaldor S.W., Kalish V.J., Davies J.F., Shetty B.V., Fritz J.E., Appelt K., Burgess J.A., Campanale K.M., Chirgadze N.Y., Clawson D.K., Dressman B.A., Hatch S.D., Khalil D.A., Kosa M.B., Lubbehusen P.P., Muesing M.A., Patick A.K., Reich S.H., Su K.S., Tatlock J.H. (1997). Viracept (nelfinavir mesylate AG1343): a potent orally bioavailable inhibitor of HIV-1 protease. J. Med. Chem..

[7] Prabu-Jeyabalan M., Nalivaika E., Schiffer C.A. (2002). Substrate shape determines specificity of recognition for HIV-1 protease: analysis of crystal structures of six substrate complexes. Structure.

[8] Heaslet H., Rosenfeld R., Giffin M., Lin Y.C., Tam K., Torbett B.E., Elder J.H., McRee D.E., Stout C.D. (2007). Conformational flexibility in the flap domains of ligand-free HIV protease. Acta Crystallogr. Sect. D.

[9] Berendsen G.H.J.C., Postma J.P.M., Gunsteren W.F.V., Hermans J., Pullman B. (1981). Interaction models for water in relation to protein hydration.

[10] Soares R.O., Batista P.R., Costa M.G.S., Dardenne L.E., Pascutti P.G., Soares M.A. (2010). Understanding the HIV-1 protease nelfinavir resistance mutation D30N in subtypes B and C through molecular dynamics simulations. J. Mol. Graph. Model..

[11] Schreiber H., Steinhauser O. (1992). Taming cut-off induced artifacts in molecular dynamics studies of solvated polypeptides. the reaction field method. J. Mol. Biol..

[12] Smith P.E., van Gunsteren W.F. (1994). Consistent dielectric properties of the simple point charge and extended simple point charge water models at 277 and 300k. J. Chem. Phys..

[13] Berendsen H.J.C., Postma J.P.M., Van Gunsteren W.F., Dinola A., Haak J.R. (1984). Molecular–dynamics with coupling to an external bath. J. Chem. Phys..

[14] Wang J., Wolf R.M., Caldwell J.W., Kollman P.A., Case D.A. (2004). Development and Testing of a General Amber Force Field. J. Comput. Chem..

[15] Onufriev A., Bashford D., Case D.A. (2004). Exploring protein native states and large-scale conformational changes with a modified generalized born model. Proteins: Struct. Funct. Bioinforma..

[16] Onufriev A., Bashford D., Case D.A. (2002). Modification of the generalized Born model suitable for macromolecules. J. Phys. Chem. B.

[17] Onufriev A., Case D.A. (2002). Effective Born radii in the generalized Born approximation: the importance of being perfect. J. Comput. Chem..

[18] Berendsen H.J.C., Postma J.P.M., Van Gunsteren W.F., Dinola A., Haak J.R. (1984). Molecular-dynamics with coupling to an external bath. J. Chem. Phys..

[19] Ryckaert J.P., Ciccotti G., Berendsen H.J.C. (1977). Numerical integration of the Cartesian equations of motion of a system with constraints: molecular dynamics of n-alkanes. J. Comput. Phys..

