# Supplementary material for: Dataset showing the impact of the protonation states on molecular dynamics of HIV protease
Source: Data Brief. 2016 Jul 25;8:1144–50. doi: 10.1016/j.dib.2016.07.040 (PMC4976645; doi:10.1016/j.dib.2016.07.040)
Supplement: Supplementary file 1 — Supplementary material [file mmc1.zip › CONFLICTS OF INTEREST STATEMENT daSilva.pdf]

CONFLICTS OF INTEREST STATEMENT

Manuscript Title:

Dataset shows the Impact of the Protonation States on Molecular Dynamics of HIV Protease

The author whose name is listed immediately below certify that she have no affiliations with or involvement in any organization or entity with any financial interest (such as honoraria; educational grants; participation in speakers' bureaus; membership, employment, consultancies, stock ownership, or other equity interest; and expert testimony or patent-licensing arrangements), or non-financial interest (such as personal or professional relationships, affiliations, knowledge or beliefs) in the subject matter or materials discussed in this manuscript.

Manuela Leal da Silva	Manuela Leal da Silva	July 3, 2016
Author Name	Author Signature	Date
